# Assessment of in vitro antimicrobial activities of ceftolozane/tazobactam and ceftazidime/avibactam against carbapenem-resistant *Pseudomonas aeruginosa* clinical isolates

**DOI:** 10.1186/s12879-025-10891-w

**Published:** 2025-04-28

**Authors:** Dalia Salem, Ahmed El-Shenawy, Heba Dahroug, Manar Zaiton, Doaa Gamal, Manal Diab

**Affiliations:** Microbiology Department, Theodor Bilharz Research Institute (TBRI), Postal Address: 30 Imbaba, P.O Box 12411, Giza, Egypt

**Keywords:** Carbapenem resistant *P. aeruginosa*, Modified carbapenem inactivation method test, Ceftazidim/avibactam, Ceftolozane/tazobactam, β-Lactam/β-Lactamase inhibitor combinations

## Abstract

**Background:**

Carbapenem resistant *Pseudomonas aeruginosa* (*P. aeruginosa*) is a global health concern that poses a challenge to treat in health care facilities. Ceftazidim/avibactam and ceftolozane/tazobactam have a potential role in treatment of multi-drug resistant phenotypes including carbapenem resistant *P. aeruginosa.* Therefore, we aimed to assess the in vitro antimicrobial activity of ceftazidime/avibactam and ceftolozane/tazobactam against carbapenem-resistant *P. aeruginosa* (CRPA) strains with different β-lactamase/carbapenemase genes.

**Methods:**

Sixty CRPA isolates identified from clinical samples were examined for antimicrobial susceptibility including ceftazidim/avibactam and ceftolozane/tazobactam by Vitek2 compact system, and carbapenemase production by modified carbapenem inactivation method (mCIM) test and carbapenemase producing genes by polymerase chain reaction (PCR).

**Results:**

Isolates were resistant to imipenem in 96.7% and meropenem in 88.3%. of isolates. Carbapenemase production by mCIM test was 70% compared to 73.3% by (PCR).

Carbapenemase encoding genes *bla*_NDM_, *bla*_VIM_ and *bla*_OXA-48_ were detected in 60%, 41.7% and 25% respectively while *bla*_IMP_ and *bla*_KPC_ weren’t identified in this study. Among CRPA, both ceftazidim/avibactam and ceftolozane/tazobactam; were sensitive in only 11.7% of the isolates. Resistance to ceftazidim/avibactam and ceftolozane/tazobactam in isolates owning *bla*_NDM_, *bla*_VIM_, *bla*_OXA-48_ and those having combined *bla*_NDM_, *bla*_VIM_ and *bla*_OXA-48_ carbapenemase resistance genes were 97.2%, 92%, 100% and 100% respectively.

**Conclusion:**

Modified carbapenem inactivation method test gave satisfactory results and could be used as an alternative to expensive genotypic methods. Ceftazidim/avibactam and ceftolozane/tazobactam were unsuccessful against carbapenem resistant *P. aeruginosa* isolates carrying carbapenemase genes especially metallo-β lactamase genes. Therefore, it is essential to detect susceptibility patterns to newly introduced β-Lactam/β-Lactamase inhibitor combinations due to the emerging resistance to these therapeutics.

## Background

*Pseudomonas aeruginosa* (*P. aeruginosa*) is one of the most important healthcare-associated opportunistic pathogens, frequently implicated in infections in critically ill or immunocompromised patients, causing pneumonia, urinary tract infections and surgical site infections [1]. Infections due to *P. aeruginosa* often pose a great therapeutic challenge, since these organisms possess intrinsic resistance to a variety of antimicrobial agents. Furthermore, *P. aeruginosa* can acquire resistance via mutation of core genes or acquisition of determinants through horizontal gene transfer of mobile genetic elements [2]*.*

Resistance to *P. aeruginosa* has been found to develop to a wide spectrum of antibiotics, including *β*-lactams, fluoroquinolones, tetracyclines, and aminoglycosides. Carbapenems have been considered as major therapeutic options for serious infections due to *P. aeruginosa*. Nonetheless, throughout the past several years, a substantial increase in carbapenem resistance of *P. aeruginosa* has drawn attention to public health systems [3]. Resistance to carbapenems in *P. aeruginosa* arises from various mechanisms, including the production of carbapenemases, over-expression of efflux pumps, loss of outer membrane porins, and the hyperproduction of extended-spectrum AmpC *β*-lactamase [4]. Beta-lactamase production frequently results in resistance to all beta-lactam antibiotics, and carbapenemase producing isolates will often exhibit multidrug resistant (MDR) phenotypes. Infection by MDR isolates restricts therapeutic options and is associated with higher risk of morbidity and mortality [5].

The most commonly detected carbapenemases in *P. aeruginosa* belong to three classes of Ambler classification*,* Ambler class A *bla*-_KPC_ and *bla*-_GES_ type. Ambler class B metallo-β-lactamases (MBLs) which are increasingly reported as a cause of high-level carbapenem resistance among *P. aeruginosa*. MBLs—encoding genes can be present on plasmids and transferred to other strains, leading to antibiotic resistant *P. aeruginosa.* Various types of MBLs are present in *P. aeruginosa* including, IMP, NDM- 1, VIM, GIM, SPM, FIM- 1, SIM, and HMB- 1 [4]. According to a meta-analysis that included 20 studies from Egypt, MBLs were the most predominant in 13 of these studies among CRPA [6]. Oxacillinases (OXAs) belong to Ambler class- D carbapenemases that were first discovered in 2003 in *Klebsiela pneumoniae* and *bla*_OXA_ genes are also carried on mobile genetic elements and transferred by plasmids.

MBLs and Oxacillinases carbapenemases production significantly alters the efficacy of therapeutics used in treatment of *P. aeruginosa* infections including ceftazidime-avibactam (CZA) and ceftolozane-tazobactam (C/T) which are novel beta lactam-beta-lactamase inhibitor combinations [7]. These combinations have broad-spectrum activity against Gram-negative bacteria, including CRPA and were approved by Food and Drug Administration (FDA) in late 2014/early 2015 for the treatment of complicated urinary tract infections and complicated intra-abdominal infections caused by *P. aeruginosa* [8].

Since, the published reports about novel β-Lactam/β-Lactamase inhibitor combinations in relation to resistance mechanisms in CRPA strains are few in Egypt, the current study aimed to (i) detect carbapenemase production among the studied *P. aeruginosa* isolates (ii) examine the distribution of carbapenemase genes; *bla*_KPC_, *bla*_NDM_, *bla*_VIM_, *bla*_IMP,_ and *bla*_OXA-48_ genes among CRPA (iii) assess the in vitro antimicrobial activity of ceftazidime/avibactam along with ceftolozane/tazobactam against CRPA strains and (iv) identify in vitro activity of ceftazidime/avibactam and/or ceftolozane/tazobactam in isolates in presence of carbapenemase genes.

## Methods

### Bacterial isolates

A total of 159 non-duplicate clinical isolates of *P. aeruginosa* were examined. Such isolates were collected from patients admitted to Theodor Bilhrz Research Institute (TBRI) hospital and outpatient clinic between 2021 and 2023. The specimens included were urine, pus, blood, sputum, and ascetic fluid. Pseudomonas strains were identified by oxidase and catalase tests and species identification was done by Vitek2 compact system (bioMérieux, France).

### Antimicrobial Susceptibility Testing (AST)

The AST was performed for all 159 Pseudomonas isolates by Kirby Bauer disk diffusion method, and Vitek2 compact system (bioMérieux, France).

#### Kirby Bauer disk diffusion method

The test was performed on Mueller Hinton agar (MHA) plates (Oxoid, France). Antimicrobial agents included imipenem 10 µg (IPM), meropenem 10 µg (MEM), ticarcillin 75 µg (TIC), ticarcillin/clavulanic 75/10 (T/C), piperacillin/tazobactam 100/10 µg (TZP), ceftazidime 30 µg (CAZ), ceftazidime/avibactam 30/20 µg (CZA), cefepime 30 µg (FEP), amikacin 30 µg (AK), gentamicin 10 µg (CN), tobramycin 10 µg (TOB), ciprofloxacin 5 µg (CIP), and aztreonam 30 µg (ATM) (Oxoid, UK). Interpretation was done according to the guidelines of the Clinical and Laboratory Standards Institute [9]*.* Isolates resistant to imipenem (IMP), meropenem (MEM) or both were selected for the study.

#### Minimum inhibitory concentration (MIC)

The (MIC) was performed using Vitek2 compact system (bioMérieux, France) for the following antimicrobials; imipenem, meropenem, ceftazidime, cefepime, piperacillin_/_tazobactam, gentamicin, amikacin, ciprofloxacin, meropenem, ceftriaxone, ceftazidime, and trimethoprim/sulfamethoxazole (TMP–/SMX)). The operation procedure was conducted according to the Vitek2 instruction manual using AES expert:2.0.0 software 9.04. GN222 cards were used and interpretation of results was according to CLSI, 2023 criteria [9]*.*

#### Minimum inhibitory concentration for ceftazidime/avibactam (CZA) and ceftolozane/tazobactame (C/T)

The (MIC) for CZA and C/T was performed by Vitek2 Compact system (bioMérieux, France) using Vitek2 AST-XN12 cards. Final concentrations of C/T from 0.25 to 32 mg/L and of CZA from 0.12 to 16 mg/L were achieved in the cards once inoculated. Breakpoints of ≤ 8 µg/ml susceptible and ≥ 16 µg/ml resistant were used for CZA, while for C/T, breakpoints of ≤ 4 mg/ml susceptible, 8 mg/ml intermediate, and ≥ 16 mg/ml resistant were applied. Breakpoints implemented were according to CLSI,2023 [9]*.*

*P. aeruginosa* ATCC 27853 was used as a quality control strain.

The tested isolates were stored at − 70 °C for further subsequent tests [10]*.*

### Detection of carbapenemase production

*P. aeruginosa* isolates showing resistance to IPM 10 µg, MEM 10 µg discs or both were selected for further phenotypic testing of carbapenemase activity and identification of carbapenemase producing genes.

#### Phenotypic detection of carbapenemase: Modified Carbapenem Inactivation method (mCIM)

The test was performed by emulsifying a loopful of 10 µl CRPA strain in 2 ml of trypticase soy broth (TSB). A 10 µg MEM disc is incubated in the former suspension for 4 h. In case of a carbapenemase-producing microorganism, the carbapenem in the disc is degraded by the carbapenemase; in contrast, if the test microorganism does not produce carbapenemase, MEM retains its antimicrobial activity after incubation in the bacterial suspension. The disc is removed from the suspension and placed onto MHA plate streaked with *E. coli* ATCC 25922 0.5 McFarland saline which is a carbapenem-susceptible indicator organism. Following overnight incubation, the zone of inhibition is measured to determine whether the MEM had been hydrolyzed (growth of the indicator organism close to the disc), or is still active (a large zone of inhibition around the disc) [9].

#### Detection of carbapenemase producing genes

All 60 carbapenem-resistant *P. aeruginosa* strains were examined for the presence of carbapenemase-encoding genes, including *bla*_KPC,_*bla*_NDM,_*bla*_VIM_, *bla*_IMP_ and *bla*_OXA-48_ using conventional PCR method.

DNA extraction for CRPA was done by boiling method [11], and DNA was stored at –20 °C until used later for conventional PCR. For *bla*_NDM_, *bla*_VIM_ and *bla*_IMP_ the reaction was performed in a final volume of 50 μL with a 2 μL of extracted DNA, 1 μL of each of the forward and reverse primers (Table 1), 25 μL of master mix maxima green master mix (ThermoScientific, USA), and the volume was completed to 50 μL by using 21 μL of sterile nuclease free water. PCR amplification was carried out in a T-personal PCR Thermal Cycler (Biometra, Uk) as follows: initial denaturation for 3 min at 94° C; followed by 35 cycles at 94° C for 45 s, annealing for each pair of primers´ temperature (Table 1) for 45 s, then at 72° C for 45 s; a final extension for 5 min at 72° C; and then maintenance at 4° C [12].

**Table 1:** PCR primers used for amplification of carbapenemase producing genes

Target gene	Nucleotide sequences (5'− 3')	Amplicon size (bp*)	Annealing temperature	Reference
*bla*_KPC_	**F: CGTCTAGTTCTGCTGTCTTG** **R: CTTGTCATCCTTGTTAGGCG**	***798***	**52 °C**	[13]
*bla*_OXA-48_	**F: GCGTGGTTAAGGATGAACAC** **R: CATCAAGTTCAACCCAACCG**	***438***	**52 °C**	[13]
*bla*_VIM_	**F; GGT CTC ATT GTC CGT GAT GGT G** **R; GGA ATC TCG CTC CCC TCT ACC T**	***242***	**61 °C**	[14]
*bla*_IMP_	**F; TCC CCA CGT ATG CAT CTG AAT TAA C** **R; CGG ACT TTG GCC AAG CTT CTA TAT T**	***258***	**60 °C**	[14]
*bla*_NDM_	**F; GGT TTG GCG ATC TGG TTT TC** **R; CGG AAT GGC TCA TCA CGA TC**	***621***	**52 °C**	[13]

*Bl*_KPC_ and *bla*_OXA-48_ were tested by multiplex reaction mixture PCR. The mix contains 2 μL of extracted DNA, 0.5 μL of each of the forward and reverse primers for both genes (a total of 2 μL), 25 μL of master mix maxima green master mix (ThermoScientific, USA), and the volume was completed to 50 μL by using 21 μL of sterile nuclease free water. Amplification was carried out with the following thermal cycling conditions: 10 min at 94 °C and 36 cycles of amplification consisting of 30 s at 94 °C, 40 s at 52 °C, and 50 s at 72 °C, with 5 min at 72 °C for the final extension [13].

Ten (10 µl) of each PCR end product were electrophoresed through a 1.5% agarose gel with 0.5 mg/ml ethidium bromide in 1 × Tris Borate EDTA buffer. The gel was visualized and photographed under ultraviolet illumination (Bio-Rad, USA) [14].

### Statistical analysis

Results were expressed as number (%). Comparison between parameters. Predictive (*P*) values < 0.05 were considered significant, < 0.01 were highly significant and < 0.001 were very high significant.

## Results

Sixty (60) (37.7%) CRPA isolates were identified out of 159 *P. aeruginosa* isolates recovered from different clinical samples. Most of the CRPA isolates, were detected from urine samples (37; 61.7%) followed by pus (9, 15%), blood (6, 10%), sputum (5, 8.3%) and ascetic fluid (3.5%). Almost all of CRPA isolates were obtained from the ICU and urology departments (49.7%, 29.8% respectively) while the outpatients represented (10%).

### Antimicrobial susceptibility patterns in CRPA isolates

Fifty-eight (58) of *P. aeruginosa* isolates (96.7%) were identified as IMP resistant, one was sensitive (1.7%), and one isolate (1.7%) showed an intermediate sensitivity. Resistance to MEM was detected in 53 (88.3%) isolates, six (10%) isolates had an intermediate sensitivity and one isolate (1.7%) was sensitive. Fifty-two isolates (86.7%) were resistant to both IMP and MEM.

**Fig. 1:**
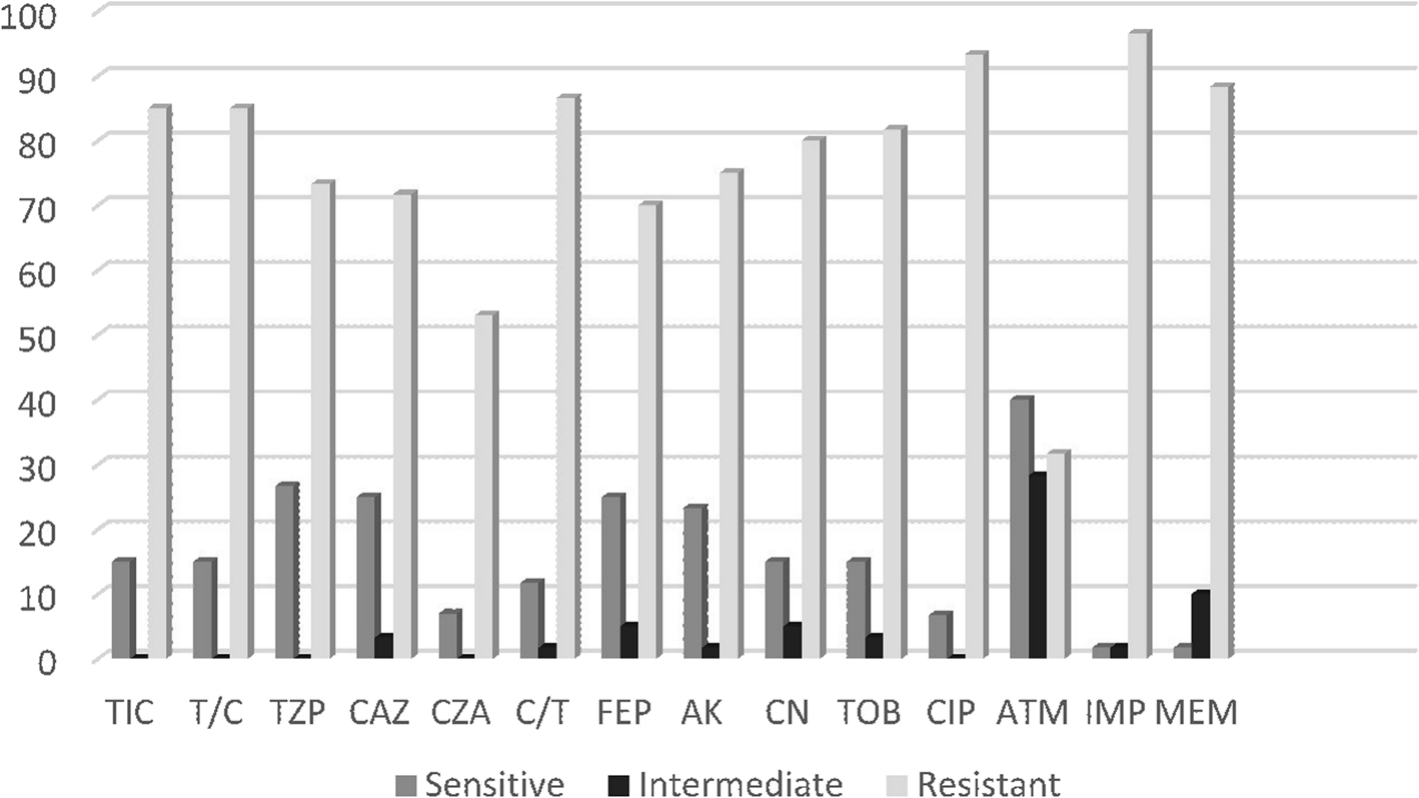
Antimicrobial susceptibility profile of the studied 60 CRPA isolates using Vitek2 compact system. TIC: Ticarcilin, T/C: Ticarcillin/Clavulanic, TZP: Piperacillin/Tazobactam, CAZ: Ceftazidim, CZA: Ceftazidim/avibactam, C/T: Ceftolozane/tazobactam, FEP: Cefepime, AK: Amikacin, CN: Gentamycin, TOB: Tobramycin, CIP: Ciprofloxacin, ATM: Aztreonam, IMP: Imipenem, MEM: Meropenem

Aztreonam remained sensitive in 40% of carbapenem non sensitive isolates, TZP was active in 26.6%, CAZ, FEP and AK were sensitive in 25%, TIC, T/C, CN, and TOB were all susceptible in 15% of tested isolates. Interestingly CZA was sensitive only in 11.7% of isolates (Fig. 1).

*P. aeruginosa* isolates resistant to IMP were also resistant to T/C in 86.2%, PEP and TZP (44/58; 75.9%), Ak (45/58; 77.6%), CN (47/58; 81.01%), TOB (48/58; 82.8%), CIP (54/58; 93.1%), FEP (42/58; 72.4%), ATM (18/58; 31.03%), CAZ (43/58; 74.1%). On the other hand, isolates resistant to MEM were also resistant to T/C in (51/53; 96.2%), PEP (44/53; 83.01%), TZP (44/53; 83.01%), Ak (41/53; 77.4%), CN (43/53; 81.1%), TOB (44/53; 83.01%), CIP (51/53; 96.2%), FEP (42/53; 79.2%), ATM (19/53; 35.8%), CAZ (43/53; 81.1%).

### Sensitivity patterns of CZA and C/T for CRPA isolates

**Fig. 2:**
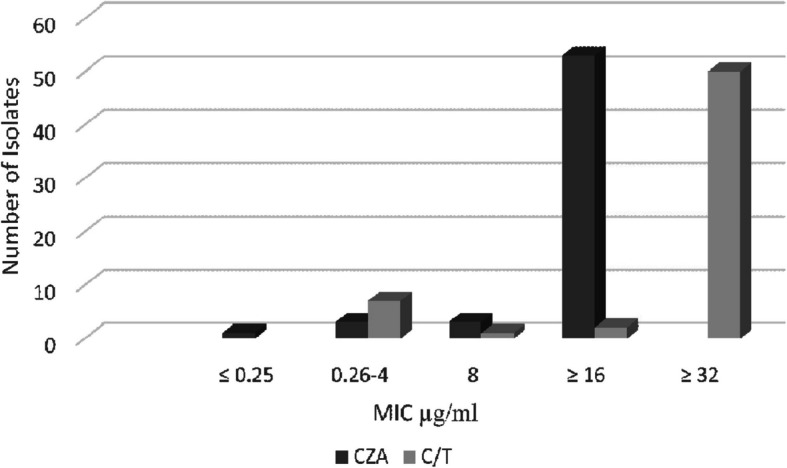
MIC distribution of ceftazidime-avibactam (CZA), ceftolozane-tazobactam (C/T), for 60 CRPA isolates. Breakpoints for (CZA) were ≤ 8 µg/ml susceptible and ≥ 16 µg/ml resistant, for (C/T) were of ≤ 4 µg/ml susceptible, 8 µg/ml intermediate, and ≥ 16 µg/ml resistant, according to CLSI, 2023

Among CRPA isolates, both CZA and C/T; were sensitive in 7/60 (11.7%), and were sensitive in only 5% of isolates simultaneously (Fig. 2).

### Detection of carbapenemase production

#### Modified carbapenem inactivation method test

Among the 60 CRPA isolates, 42 (70%) were positive (carbapenemase producers), 8 (13.3%) isolates were undetermined carbapenemase producers (to be confirmed by conventional PCR) and 10 (16.7%) were negative (non carbapenemase producer).

#### Genotypic detection of carbapenemases genes

**Fig. 3:**
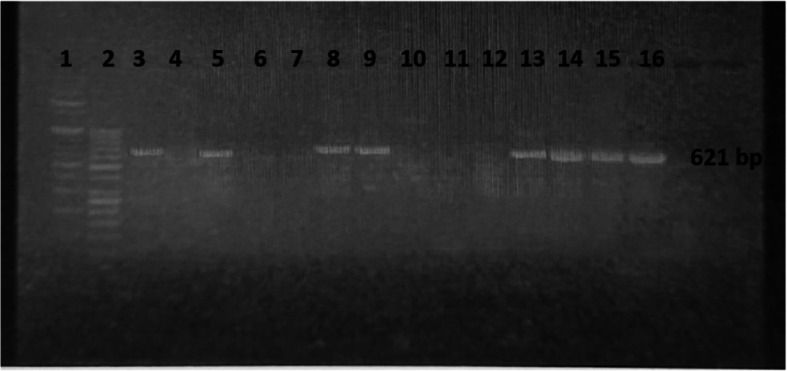
Agarose gel electrophoresis of PCR products of CRPA *bla*_NDM_ positive gene (621 bp) Lane 1: Molecular weight marker (100 bp), Lane 2: Molecular weight marker (ladder 50 bp), Lane 3: Positive control, Lane 4: Negative control, Lane (5, 8, 9,13, 14, 15,16): Positive DNA samples *bla*_NDM_ PCR amplification product

**Fig. 4:**
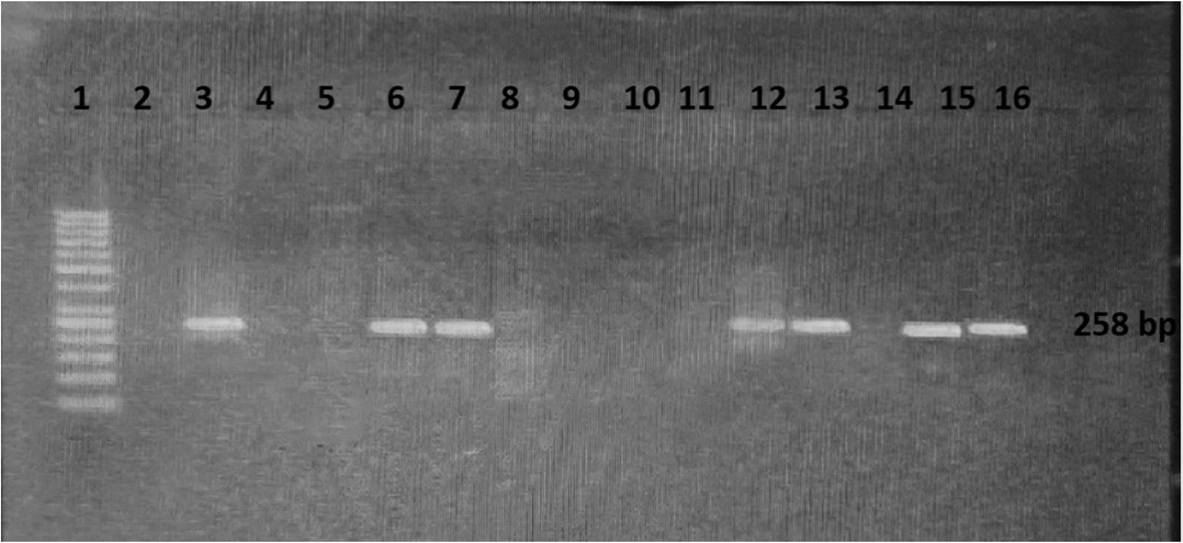
Agarose gel electrophoresis of PCR products of CRPA *bla*_VIM_ positive gene (258 bp) Lane 1: Molecular weight marker (ladder 50 bp). Lane 2: Negative control. Lane 3: Positive control. Lane (6, 7, 12, 13, 14, 15,16): Positive DNA samples *bla*_VIM_ PCR amplification product

Screening of carbapenemases genes (*bla*_KPC_, *bla*_NDM,_*bla*_VIM_, and *bla*_IMP_, and *bla*_OXA-48_) revealed that at least one gene was found in (44; 73.3%) of all tested 60 CRPA strains. *Bla*_NDM_ was the most frequently detected gene in (36, 60%) of strains, *bla*_VIM_ was identified in (25, 41.7%), *bla*_*OXA*−48_ was recognized in (15, 25%), while *bla*_IMP_ and *bla*_KPC_ weren’t detected in any of the tested strains. Both *bla*_NDM_ and *bla*_VIM_ were found simultaneously in (17; 28.3%) of strains, whereas *bla*_NDM,_*bla*_VIM_ and *bla*_*OXA*−48_ combined were identified in (5; 8.3%) of the tested strains (Figs. 3 and 4).

Each one of *bla*_NDM_ and *bla*_VIM_ genes were found solely in 10/36 (27.8%) and 4/25 (16%) respectively, while *Bla*_*OXA*−48_ was constantly associated with either *bla*_NDM_, *bla*_VIM_ or both.

Forty-two (70%) isolates were carbapenemase producers by mCIM test, eight strains (13.3%) were indeterminate carbapenemase producers and 10 (16.7%) were non carbapenemase producers.

The mCIM test was a good negative predictive test as nine out of ten (90%) isolates that were non carbapenemase producers by mCIM test were also negative for *bla*_KPC,_*bla*_NDM,_*bla*_VIM,_*bla*_IMP_ and *bla*_OXA-48_ genes. Among the 42 positive mCIM CRPA strains, 41 (97.6%) had at least one of the carbapenemase genes. Only one strain (1.7%) was carbapenemase producer by mCIM test while *bla*_NDM,_*bla*_VIM_ and *bla*_OXA-48_ genes were negative, but this doesn’t exclude the presence of other carbapenemase producing genes that weren’t tested in the study. Confirmed carbapenemase production by PCR (isolates harboring either *bla*_NDM,_*bla*_VIM_ or *bla*_OXA-48_ genes) was detected in 44 isolates, in which mCIM test was positive in 41 (93.2%) strains and the remaining three (6.8%) carbapenemase producing isolates showed two (4.8%) indeterminate, and one (2.4%) had negative mCIM result (Table 2).

**Table 2:** Performance characteristics of mCIM test for detection of carbapenemase production in 60 CRPA strains

Carbapenemase genes by PCR (No. 44)	mCIM Test					
	**Positive** **No. (42)**		**Indeterminate** **No. (8)**		**Negative** **No. (10)**	
	**No**	**%**	**No**	**%**	**No**	**%**
**NDM**						
**Positive** (36)	36	100	0.0	0.0	0.0	0.0
**Negative** (24)	6	25	8	33.3	10	41.7
**VIM**						
**Positive** (25)	22	88	2	8	1	4
**Negative** (35)	20	57.1	6	17.1	9	25.7
**OXA**						
**Positive** 15	15	100	0.0	0.0	0.0	0.0
**Negative** 45	27	60	8	17.8	10	22.2
**NDM & VIM Positive** (17)	17	100	0.0	0 0.0	0.0	0.0
**NDM & OXA**						
**Positive (13)**	13	100	0.0	0.0	0.0	0.0
**VIM & OXA**						
**Positive (7)**	7	100	0.0	0.0	0.0	0.0
**NDM or VIM or OXA**						
**Positive** (44)	41	93.2	2	4.5	1	2.3
**NDM & VIM & OXA**						
**Negative** (16)	1	6.25	6	37.5	9	56.25

Data are expressed as number (%)

Carbapenemases genes were absent in 16/60 (26.7%) of the CRPA studied isolates, where mCIM was indeterminate in 6/16 (37.5%), and showed negative result in 9/16 (56.25%), and only one isolate was carbapenemase producer by mCIM (Table 2).

Among the 8 strains that showed indeterminate carbapenemase production by mCIM, 2/8 (25%) harbored *bla*_VIM_ genes (Table 2).

Resistance to CZA and C/T in isolates owning *bla*_NDM_, *bla*_VIM_, *bla*_OXA_, both *bla*_NDM_ and *bla*_VIM,_ as well as those having all three genes (*bla*_NDM_, *bla*_VIM_, *bla*_OXA_) carbapenemase resistance genes were 97.2%, 92%, 100%, 100% and 100% respectively. Alternatively, in absence of carbapenemase genes, resistance was relatively lower reaching 75% for CZA and 68.8% for C/T. Sensitivity to both drugs were the same (25%) in absence of resistance genes (Table 3).

**Table 3:** Association between CZA and C/T resistance and types of carbapenemase genes among 60 CRPA strains

Carbapenemase Genes		CZA		
		**Sensitive** **No. (7)**	**Indeterminate** **No. (0)**	**Resistant** **No. (53)**
**NDM**	**Positive No. (36)**	1 (2.8%)	0 (0.0%)	35 (97.2%)
	**Negative No. (24)**	6 (25%)	0 (0.0%)	18 (75%)
**VIM**	**Positive No. (25)**	2 (8%)	0 (0.0%)	23 (92%)
	**Negative No. (35)**	5 (14.3%)	0 (0.0%)	30 (85.7%)
**OXA**	**Positive** **No.(15)**	0.0 (0%)	0.0 (0%)	15 (100%)
	**Negative** **No. (45)**	7 (15.6%)	0.0 (0%)	38(84.4)
**NDM & VIM**	**Positive** **No. (17)**	0 (0.0%)	0 (0.0%)	17 (100%)
	**Negative** **No. (16)**	4 (25%)	0 (0.0%)	12 (75%)
**NDM & OXA**	**Positive** **No. (13)**	0 (0.0%)	0 (0.0%)	13 (100%)
	**Negative** **No.(22)**	6 (27.3%)	0 (0.0%)	16 (72.7%)
**VIM & OXA**	**Positive** **No. (7)**	0 (0.0%)	0 (0.0%)	7(100%)
	**Negative** **No. (27)**	5(18.5%)	0 (0.0%)	22(81.5%)
**NDM &** **VIM** **&** **OXA**	**Positive** **No. (5)**	0 (0.0%)	0 (0.0%)	5(100%)
	**Negative** **No. (16)**	4(25%)	0 (0.0%)	12(75%)
**Carbapenemase Genes**		**C/T**		
		**Sensitive** **No. (7)**	**Indeterminate** **No. (1)**	**Resistant** **No. (52)**
**NDM**	**Positive** **No. (36)**	1 (2.8%)	0 (0.0%)	35 (97.2%)
	**Negative** **No. (24)**	6 (25%)	1 (4.2%)	17 (70.8%)
**VIM**	**Positive** **No. (25)**	2 (8%)	0 (0.0%)	23 (92%)
	**Negative** **No. (35)**	5 (14.3%)	1 (2.8%)	29 (82.9%)
**OXA**	**Positive** **No.(15)**	0 (0.0%)	0 (0.0%)	15 (100%)
	**Negative** **No.(45)**	7 (15.6%)	1 (2.2%)	37 (82.2%)
**NDM & VIM**	**Positive** **No. (17)**	0 (0.0%)	0 (0.0%)	17 (100%)
	**Negative** **No. (16)**	4 (25%)	1 (6.25%)	11 (68.75%)
**NDM &** **OXA**	**Positive** **No.(13)**	0 (0.0%)	0 (0.0%)	13 (100%)
	**Negative** **No.(22)**	6 (27.3%)	1 (4.5%)	15(68.2%)
**VIM&** **OXA**	**Positive** **No.(7)**	0 (0.0%)	0 (0.0%)	7 (100%)
	**Negative** **No.(27)**	5 (18.5%)	1 (3.7%)	21 (77.8%)
**NDM & VIM & OXA**	**Positive** **No.(5)**	0 (0.0%)	0 (0.0%)	5 (100%)
	**Negative** **No.(16)**	4 (25%)	1 (6.25%)	11 (68.8%)

Data are expressed as number (%)

## Discussion

Carbapenem resistant *P. aeruginosa* is a global public health concern associated with reduced therapeutic options, prolonged hospital stays and increased mortality [15]*.* Epidemiology of MDR infections across the Arab League has shown that Egypt displays resistance levels reaching 51% of CRPA [2]*.* In the current study, CRPA recovered from clinical samples at TBRI hospital accounted for a rate of 37.7% and 46.7% of them were from ICU. In Egypt, *El-Sokkary *et al*.* [16] reported a rate of 30.4% of CRPA, while a higher prevalence of 59% was detected by *Basha and colleagues* [17]. An alarming prevalence of 96.9% [18] and 98.2% in a recent study were also detected [19]. A comparative incidence of resistance was detected in Saudi Arabia by *Khater and AlFaki* [20]. Global data showed variation in the incidence according to geographical distribution. In Pakistan the prevalence rate of CRPA was 44% [21]*,* in Turkey a pooled prevalence of 30.1% resistance against meropenem and 28.0% for imipenem was found [22].

Surveillance of antimicrobial resistance in Europe showed that the incidence of CRPA was below 5% in two (4%) of the 45 countries reporting data, whereas six (13%) countries reported percentages equal to or above 50% [23]. In the United States, 10–30% of *P. aeruginosa* isolates are carbapenem-resistant [24]*.* In China meropenem resistant *P. aeruginosa* showed higher prevalence of (64.7%) [25]*.*

Our results revealed that the main recovery department of CRPA was the ICU (46.7%), which was in accordance with numerous studies that pointed out the high prevalence of CRPA isolated from ICU compared to other departments of the hospital [26, 27]*.* This may be due to high antibiotic prescribing practices at Critical Care Units. *P. aeruginosa* isolates resistant to carbapenems were mainly isolated from urine samples (61.7%), such result was in agreement of other previous study (60%) from Egypt [28].

We have noticed that most of *P. aeruginosa* clinical isolates exhibited high level of resistant rates to the majority of the used antibiotics. The top antibiotic with highest resistant rate was ciprofoxacin (93.3%), followed by ticarcillin/clavulanic, amikacin and piperacillin/tazobactam (85%, 75% and 73.3% respectively). Aztreonam was among the least antibiotics showing resistance 31.7% as it evades hydrolytic disintegration by MBLs. This was totally different from the pattern of resistance detected by *Elsawy *et al. [29] as resistance to ciprofloxacin was 25%, amikacin 4.2%, piperacillin/tazobactam 8.3%, and aztreonam showed a resistance rate of 45.8%. Our results were comparable to those obtained by *Khater and AlFaki* [20] in Saudi Arabia as ciprofloxacin showed 100% resistance, amikacin 90.6%, piperacillin/tazobactam 81.2%, and aztreonam exhibited a resistance of 21.8%. In Iran the highest resistance was found to ceftazidim (91.6%), piperacillin/tazobactam was the least resistant, while gentamycin, amikacin, tobramycin, cefepime, piperacillin, ciprofloxacin and aztreonam all gave a resistance rate ranging 83.2%− 88.8% [13]. Such different resistant rates could be due to variations in antibiotic consumption in different countries.

Although β-Lactam/β-Lactamase inhibitor combinations are relatively newly introduced, and are saved to MDR organisms, resistance has been detected, and has been documented in *P. aeruginosa* [25]*.* In the current study, both CZA and C/T exhibited the same sensitivity (11.7%) against CRPA isolates, while resistance was 88.3% and 86.6% respectively. A possible explanation is that most of carbapenemases detected in this study belong to the metallo β-lactamases (73.3%) NDM genotype (60%)*. Schaumburg and colleagues* [30] reported that carbapenem resistance in Pseudomonas strains is associated with resistance to CZA in 50.9%. Moreover, CZA is less effective for MβL-positive strains, showing the highest resistance rate (> 95%), as class B β-Lactamases can hydrolyze all clinically used serine β-lactamase inhibitors, including avibactam [31]. Our results aligned with those of a previous study in Egypt, as sensitivity for both CZA and C/T was 23.5%. This is mainly attributed to the abundance of *bla*_NDM_ in their study (66.7%) [5]*.*

*Nasser *et al. [32] reported higher susceptibility rates to CZA and C/T, where antimicrobial resistance is attributed mainly to causes other than carbapenemase production, as efflux pump and decreased membrane permeability by down regulation of membrane porins. In Turkey, *Hazirolan and Ozkul* [4] demonstrated that sensitivity to CZA and C/T were 62.4% and 55.1% respectively, where only 24.5% of isolates produced MβL. A surveillance in western Europe reported that 72% of MDR *P. aeruginosa* were C/T susceptible considering that only 8.8% were MβL [33]. In a three center study in USA, both antimicrobial agents were active against > 80% of β-lactam-resistant *P. aeruginosa* isolates since most of the isolates had *oprD* mutations indicating decreased outer membrane permeability of the drugs [34]*.* We can suggest that, the variation in the activity of CZA and C/T among *P. aeruginosa* strains in different regions of the world might reflect different resistance mechanisms expressed by such organisms, thus highlighting how these mechanisms can have different impacts on the efficacy of the studied combinations.

It is extremely important to implement a rapid reliable method to detect carbapenemase production to avoid delay or inaccurate treatment of patients. Detection of carbapenemase production by mCIM test was a valuable method giving accurate results with low cost even where metallo-β-lactamases were mostly detected in this work. The test was positive in 70% compared to 73.3% detected by conventional PCR as the gold standard. The specificity of the test was excellent (90%) as ninety percent of isolates that showed negative mCIM test were all negative for carbapenemase production with PCR. Therefore, it is a good negative predictive test. Only one isolate gave a false positive result (was positive by mCIM and negative by PCR. This could be due to the production of a carbapenemase other than the studiedgenes.

*Lisbao *et al*.* [35] reported that mCIM offers an alternative method for carbapenemase detection in *P. aeruginosa*. Concordance of mCIM test results and conventional PCR of carbapenemase resistance genes reached up to 87%, although it has limitations for the Ambler class A and B enzymes. In this study, three out of 44 isolates gave discrepant results between conventional PCR and mCIM test. These strains were positive by PCR (all 3 belong to *bla*_VIM_) but gave two indeterminate and one negative result by mCIM test. Inconsistent results between PCR and mCIM test were also detected in a previous study mainly in isolates harboring *bla*_GES_; an Ambler group A carbapenemase; and to a lesser extent in *bla*_NDM_ and *bla*_VIM_ carbapenemases [35]*.* The mCIM test has its liabilities as the test requires an overnight incubation in contrast to molecular methods, in which results can be obtained within hours. Moreover, a positive mCIM result does not provide information about the type of carbapenemase present in a given bacterial isolate [36]*.*

Worldwide, the dissemination of carbapenemases in *P. aeruginosa* is greatly variable with MBLs remaining the most predominant [37]*.* VIMs were the most widely distributed (Middle East, South America, Africa), followed by IMPs and NDMs [38]*.* However, ATLAS of Global surveillance program reported the shift in predominance of carbapenemase resistance genes in carbapenem resistant Enterobacterales (CRE) and CRPA from VIM as the predominant carbapenemase (14.7%) in Africa and Middle East between 2017–2019 to NDM between 2018–2022 [39].

The *bla*_NDM_ was the most common detected carbapenemase (60%) followed by *bla*_VIM_ (41.7%) by conventional PCR, and both were found simultaneously in 28.3%. *Bla*_Oxa-48_ was carried by in 25% of isolates. *Bla*_IMP_ and *bla*_KPC_ weren’t detected in any of our clinical isolates. A recent study from Egypt revealed comparable results [5]. *Basha and colleagues* [17]*,* confirmed that *bla*_NDM_ was the predominant carbapenemase genes (90.9%) followed by *bla*_VIM_, (18.8%) in Egypt. In addition, they also could not reveal *bla*_IMP_ in their study. In addition, a recent study by *Afify and colleagues* [19] revealed comparable results regarding *bla*_NDM_ and *bla*_OXA-48_ carbapenemases as they were prevalent in 55% and 15% of the isolates respectively, *bla*_VIM_ was present in only 5% of CRPA isolates, *bla*_IMP_ and *bla*_KPC_ weren’t also detected in their studied isolates. The absence of *bla*_KPC_ and *bla*_IMP_ is consistent with studies describing the scarcity of those genetic determinants in the region [40, 41].

The phenotypic mCIM test could provide early evidence about carbapenemase production which can guide clinicians to start β-Lactam/β-Lactamase inhibitor combinations mainly as they are highly active against non carbapenemase producing CRPA [7]*.* Unfortunately, this wasn’t applicable in this study considering that only two mCIM negative isolates (2/10; 20%) were sensitive for CT, and another two (20%) were sensitive for CZA. A possible cause could be the presence of another mechanism of resistance as over expression of efflux pump mechanism [42]. Better efficacy in such case could be expected by the novel drug cefiderocol and tigecyclin [43], as cefiderocol showed clinical success rates of treatment reaching 84.3% in infections caused mainly by *P. aeruginosa* resistant to CZA and C/T [44], which could be a major breakthrough in treatment of MDR infections.

## Conclusion

The high prevalence of carbapenem resistant *P. aeruginosa* in our hospital with predominance of MBL producing isolates mainly of the NDM genotype, enhances the value of detection of carbapenem resistance and the mechanism of such resistance for treatment of patients suffering from comparable infections. Detection of carbapenemase production by the promising phenotypic mCIM test could be an alternative to expensive genotypic methods as it is an accurate, easy and cheap test. It is essential to detect susceptibility patterns to newly introduced β-Lactam/β-Lactamase inhibitor combinations as ceftazidim/avibactam and ceftolozane/tazobactam due to emerging resistance to these therapeutics. Our data showed that ceftazidim/avibactam and ceftolozane/tazozobactam were unsuccessful in treating carbapenem resistant *P. aeruginosa* carrying metallo-β carbapenemases, therefore, novel and more effective therapeutics are urgently needed for treating patients with such superbugs isolates.

## Data Availability

All data generated or analyzed during this study are included in this published article.

## Abbreviations

*P*. *aeruginosa*: *Peudomonas* *aeruginosa*
MβLs: Metallo-beta lactamases
CRPA: Carbapenem resistant Pseudomonas aeruginosa
MDR: Multidrug resistant
TBRI: Theodor Bilharz Research Institute
mCIM: Modified carbapenem inactivation method
PCR: Polymerase chain reaction
CLSI: Clinical and Laboratory Standards Institute
IPM: Imipenem
MEM: Meropenem
TIC: Ticarcillin
T/C: Ticarcillin/clavulanic
TZP: Piperacillin/tazobactam
FEP: Cefepime
AK: Amikacin
CN: Gentamycin
TOB: Tobramycin
CIP: Ciprofloxacin
ATM: Aztreonam
CZA: Ceftazidim/avibactam
C/T: Ceftolozane/tazobactam
TSB: Trypticase soy broth
MHA: Mueller Hinton agar
MIC: Minimum inhibitory concentration
